# Clinical and Molecular Features of Early Infantile Niemann Pick Type C Disease

**DOI:** 10.3390/ijms21145059

**Published:** 2020-07-17

**Authors:** Berna Seker Yilmaz, Julien Baruteau, Ahad A. Rahim, Paul Gissen

**Affiliations:** 1 Genetics and Genomic Medicine Department, Great Ormond Street Institute of Child Health, University College London, London WC1N 1EH, UK; j.baruteau@ucl.ac.uk (J.B.); p.gissen@ucl.ac.uk (P.G.); 2 Department of Paediatric Metabolic Medicine, Mersin University, Mersin 33110, Turkey; 3 National Institute of Health Research Great Ormond Street Biomedical Research Centre, London WC1N 1EH, UK; 4 Metabolic Medicine Department, Great Ormond Street Hospital for Children NHS Foundation Trust, London WC1N 3JH, UK; 5 UCL School of Pharmacy, University College London, London WC1N 1AX, UK; a.rahim@ucl.ac.uk

**Keywords:** Niemann Pick disease type C, early infantile onset, neurological manifestations

## Abstract

Niemann Pick disease type C (NPC) is a neurovisceral disorder due to mutations in *NPC1* or *NPC2*. This review focuses on poorly characterized clinical and molecular features of early infantile form of NPC (EIF) and identified 89 cases caused by *NPC1* (NPC1) and 16 by *NPC2* (NPC2) mutations. Extra-neuronal features were common; visceromegaly reported in 80/89 NPC1 and in 15/16 NPC2, prolonged jaundice in 30/89 NPC1 and 7/16 NPC2. Early lung involvement was present in 12/16 NPC2 cases. Median age of neurological onset was 12 (0–24) and 7.5 (0–24) months in NPC1 and NPC2 groups, respectively. Developmental delay and hypotonia were the commonest first detected neurological symptoms reported in 39/89 and 18/89 NPC1, and in 8/16 and 10/16 NPC2, respectively. Additional neurological symptoms included vertical supranuclear gaze palsy, dysarthria, cataplexy, dysphagia, seizures, dystonia, and spasticity. The following mutations in homozygous state conferred EIF: deletion of exon 1+promoter, c.3578_3591 + 9del, c.385delT, p.C63fsX75, IVS21-2delATGC, c. 2740T>A (p.C914S), c.3584G>T (p.G1195V), c.3478-6T>A, c.960_961dup (p.A321Gfs*16) in *NPC1* and c.434T>A (p.V145E), c.199T>C (p.S67P), c.133C>T (p.Q45X), c.141C>A (p.C47X) in *NPC2*. This comprehensive analysis of the EIF type of NPC will benefit clinical patient management, genetic counselling, and assist design of novel therapy trials.

## 1. Introduction

Niemann Pick type C (NPC) disease (MIM#257220 and MIM#607625) is a progressive, irreversible, and debilitating neurovisceral lysosomal storage disorder characterized by impaired intracellular lipid trafficking, which leads to the accumulation of unesterified cholesterol, sphingosine, and a range of glycosphingolipids in the endolysosomal compartment [1,2].

Bi-allelic mutations in one of the two genes cause NPC: *NPC1* (MIM*607623) in 95% of cases and *NPC2* in the remainder (MIM*607625) [1,3,4]. NPC1 (encoded by *NPC1*) is a large 1278 amino acid transmembrane protein localized to late endosomes and lysosomes. It contains three luminal and 13 transmembrane domains, as well as a lysosomal signal region [5]. The luminal domains are highly glycosylated and have a cholesterol-binding region and a sterol-sensing domain (SSD) [6,7]. A soluble, cholesterol-binding protein, NPC2, is made up of 151 amino acids and four highly conserved domains responsible for cholesterol- binding and release [8,9,10]. Based on recent studies, the dynamic interface between NPC2 and NPC1 proteins facilitates the cholesterol transport by reducing the energy barrier and stabilizing the passage [11].

The estimated incidence of this devastating disease is approximately 1/100,000 births, with variations between ethnic groups [12]. Higher incidence has been reported in some isolated populations such as Acadians in Nova Scotia, Hispanics from Southern Colorado and New Mexico, and Greeks on a small Aegean island due to a founder effect [1,13,14,15]. It is also suggested that the adult-onset form may have a higher prevalence at 1/19,000–36,000 [16]

The clinical spectrum and progression of disease are extremely heterogeneous [17,18]. Disease onset occurs across the lifespan, from the prenatal period to adulthood and a range of visceral, neurological, and psychiatric clinical features seem to appear and progress differently in individual patients [1,3,12,17,19,20]. The age of onset of neurological symptoms determines the speed of progression of the disease and allows life expectancy predictions to be made [1]. Hence, NPC is best classified according to the age of onset of neurological manifestations as follows:

1. Visceral-neurodegenerative form
  - Early-infantile (neurological onset <2 years)
2. Neurodegenerative form
  - Late-infantile (neurological onset 2–6 years)
  - Juvenile (neurological onset 6–15 years)
3. Psychiatric-neurodegenerative form
  - Adult (neurological onset >15 years) [12]

More than 40% of patients with all forms of NPC present in the first year of life with cholestatic jaundice and visceromegaly, usually hepatosplenomegaly [1,17]. Although jaundice and hepatomegaly disappear after 6–12 months in most patients, splenomegaly usually remains [20,21]. Very infrequently (probably <5% of the total NPC population) patients present in the first 2 weeks of life with liver failure leading to death unless liver transplantation is performed [22]. In such rare cases patients may recover after liver transplant and have a variable period of reasonable health before succumbing to neurological disease [23,24,25,26]. Moreover, there is another distinct phenotype, known as fetal onset NPC which presents with fetal ascites/nonimmune hydrops fetalis [1,12,27,28,29].

In the majority of patients, neurological symptoms start insidiously after a varying period of normal or slightly delayed development. While initial symptoms are often nonspecific such as hypotonia, developmental delay, and clumsiness, regression gradually occurs with patients experiencing loss of motor skills and cognitive decline [1,17,30]. Vertical supranuclear gaze palsy, cataplexy, or drop attacks could occur early in the presentation of NPC. Ataxia, dysphagia, dysarthria, and loss of cognitive skills demonstrate progression of the disease and psychiatric symptoms are typical in adult patients [4,31,32,33]. Patients with early onset NPC have a more severe and rapidly progressing course than those with later onset of neurological disease [1,12,27,34,35].

Whilst the features and rate of progression of the common juvenile form of NPC are well known, the specific disease characteristics of the rarer early infantile form (EIF) is not well established. The dearth of knowledge in this area impairs provision of accurate prognosis for the families and potentially inhibits design of clinical trials for novel therapies. Thus, in this article we aimed to provide better insight into this form of the disease on the basis of our review of published cases.

## 2. Results

### 2.1. Literature Search

**Figure 1:**
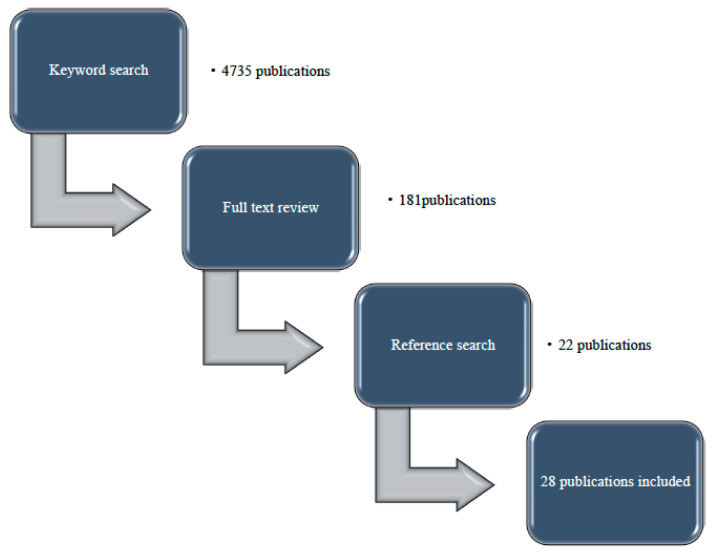
Flowchart of the selection process for the publications included.

Keyword “Niemann Pick Disease type C” yielded 1097, keyword “Niemann Pick Disease” gave 3638 publications. A total of 181 articles underwent full text review. According to the reference list check 22 additional articles were assessed and in 28 of publications patients met our inclusion criteria. Articles included in this study were published between 1988 and 2020. Figure 1 shows a flowchart of the search process for the publications included in this study.

### 2.2. Early Infantile Cases with NPC1 Mutations

Several international publications reported clinical and molecular findings in their cohorts of NPC cases (Table 1). In the Czech Republic, the estimated prevalence of NPC was suggested as 0.93 per 100,000 births [36] and 56 NPC patients, 30 (54%) females and 26 (46%) males, were reported in an observational, retrospective analysis of all NPC cases, diagnosed between 1975 and 2012. A total of 21 patients (38%) from 10 families were relatives; siblings and cousins. A total of 55 patients were diagnosed with NPC1 and one was diagnosed with NPC2. A total of 8 of 55 NPC1 patients (14.5%) had EIF (six *NPC1*, 1 *NPC2* mutations and two with unknown genetics) [36]. Psychomotor retardation or regression between 6 months and 2 years of age were the most common symptoms among EIF patients. All of the EIF patients had visceral symptoms including neonatal hepatosplenomegaly and/or prolonged neonatal jaundice. Ataxia and speech retardation occurred around 2 years of age and was followed by gelastic cataplexy, seizures, oculomotor abnormalities, dysphagia, and spasticity. Most of these patients deceased around 5 years of age due to respiratory complications [36].

The prevalence of NPC in the UK is estimated as 0.78 per 100,000 birth [37]. An observational retrospective study revealed a total of 146 NPC patients born between 1954 and 2009, among whom 77 (53%) were female and 69 (47%) male [37]. A total of 112 patients (77%) had at least one identified disease-causing mutation; 110 had *NPC1* mutations and two had *NPC2* mutations. Six patients (4%) had the visceral neonatal form, eight patients (5%) had EIF, 51 patients (35%) had LIF, 42 (29%) had juvenile and 25 (17%) had adolescent/adult phenotype [37]. The mean (SD; range) age at onset of neurological manifestations in EIF group was 1.1 (0.7; 0–2.0) years. Moreover, the mean (SD) time between onset of neurological manifestations and diagnosis was 0.26 (1.49) years, with diagnostic testing based on detection of visceral symptoms in four patients. The median age at death was 65 months, ranged 40–101 months in this group which was reported as death related to NPC. A total of 6/8 patients (75%) exhibited both prolonged neonatal jaundice and hepatosplenomegaly. Most frequent neurological symptoms were developmental delay, ataxia, and dysarthria which were developed in all eight patients. Cataplexy/epileptic seizures and swallowing difficulties were each recorded in 6/8 (75%) patients. Ophthalmic assessments revealed vertical supranuclear gaze palsy (VSGP) in 5/8 (63%) of EIF patients [37].

The International NPC registry, a prospective observational cohort study, included 163 NPC patients from 14 European countries, Australia, Brazil, and Canada and 137 patients had available genetic results; 134 had *NPC1* and three had *NPC2* mutations. A total of 16 patients (11%) were in EIF group [2]. Almost all EIF patients had visceromegaly and/or prolonged jaundice and eight out of 16 (50%) were diagnosed before the appearance of neurological manifestations. Three patients in the EIF onset group presented with perinatal hypotonia. Developmental delay, dysphagia, and VSGP were the most common neurological manifestations among the EIF cases [2].

A total of 40 Spanish NPC1 patients diagnosed between 1988 and 2003 were presented in one study [34] where the only available clinical data was the presence of visceromegaly and neonatal jaundice. Though the publication lacked expanded neurological information, as many as 12/40 (30%) patients were determined as “severe infantile” with a neurological presentation in the first 2 years of life [38].

Similar to the above study, 30 NPC1 patients who were referred for molecular analysis from Spain were described in another report. According to the age of neurological onset, three (10%) neonatal, 10 (33.3%) EIF, six (20%) LIF, six (20%) juvenile, and two (6.6%) adult cases were noted [39].

In a multicenter study from Italy, 44 NPC patients were reported. A total of 41/44 patients had *NPC1* mutations and 11/41 (26.8%) were in the EIF group [40]. A total of three NPC1 cases who died during the first month of life due to liver or respiratory insufficiency without signs of neurological involvement were labelled as early infantile systemic lethal form (EISL) [40].

In a more recent updated publication, 105 patients from 83 unrelated families were included in a collaborative multicenter study aimed at characterizing the molecular bases of Niemann–Pick C in Italy [41]. Clinical phenotypes were classified according to the age at onset of neurological symptoms and 97/105 cases had *NPC1* mutations and 21.9% of these NPC1 cases were EIF patients [41].

A prospective, open-label study reported all pediatric NPC patients treated with miglustat in France between October 2006 and December 2010, adult cases were not included. A total of 19/20 cases were NPC1 patients, and 8/19 (42%) had EIF presentation [42]. A history of hepatosplenomegaly and/or neonatal jaundice was recorded in all eight EIF patients and liver biopsy revealed evidence of cirrhosis in two patients [42]. In EIF group, neurological symptoms including hypotonia, developmental delay and swallowing difficulties were first detected between 5 and 12 months of age [42]. VSGP was observed at 9, 18, and 24 months of age in five patients, but no patients had cataplexy [42]. One patient had significant dysphagia at 5 months of age, requiring enteral feeding with nasogastric tube, gastrostomy was inserted at the age of 9 months [42]. Four patients had peripheral neuropathy and distal motor deficit [42].

A total of 23 NPC1 patients were reported from Egypt in a study designed to describe the spectrum of clinical, biochemical and molecular profile of the disease [43]. Disease onset was reported as neonatal in eight patients (presenting <3 months of age), EIF in six (presentation from 3 months–2 years of age), LIF in three (2–6 years), and juvenile in six patients (6–15 years), nevertheless, adult patients were not reported in this cohort [43]. Nineteen patients were offspring of consanguineous marriages (82.6%), while positive family history was reported in 13 families (65%) [43]. Age of neurological onset varied between 8 and 18 months in the EIF group while all of these cases had also visceral symptoms [43].

A total of 21 NPC patients diagnosed between 2009 and 2012 were included in an observational study from Iran [41]. All patients were from consanguineous parents and three of them had EIF. Among EIF and LIF patients, 70% had hepatomegaly with or without spleen involvement and 40% had prolonged neonatal jaundice as a presenting feature. Neurodevelopmental delay was seen in all three patients with EIF [44]. In another Iranian cohort, 11 NPC patients were reported. A total of 5/11 were EIF patients with accompanying visceromegaly and three of them died in the first 2 years of life [45].

A publication from China reported that 7/12 total NPC cohort had EIF disease [42]. A total of 6/7 had *NPC1* mutations, 4/6 had splenomegaly, and 2/6 had hepatosplenomegaly. Frequent falls and developmental delay were the most common neurological symptoms [46].

A cross-sectional analysis of 42 NPC patients residing in Germany or Switzerland were designed to assess neuropsychiatric symptoms. A total of 6/42 patients had a neurological onset in the first 2 years of life, five of whom had *NPC1* mutations [21].

A retrospective study from Israel reported 12 patients from six nuclear families of Bedouin origin. A total of 5/12 patients had EIF with *NPC1* mutations and all of them died before 5 years of age [47].

Overall, we identified 89 cases reported as EIF NPC due to *NPC1* mutations (Table 2). There were 40 (45%) females and 23 (26%) males and 26 (29%) of unknown gender. While the exact age of neurological symptom onset was included in 66 cases, 23 cases were only determined as “early infantile Niemann Pick type C”. In these 66 cases, median age of neurological onset was 12 months (range 0–24 months). Positive family history was noted in 27 patients and there were five pairs of siblings. Consanguinity status was mentioned in 35 cases and 30 of them were from consanguineous parents.

**Figure 2:**
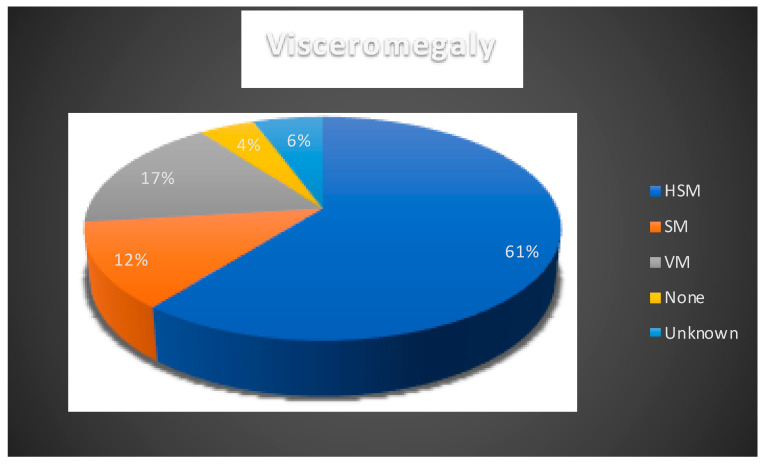
Presence of visceromegaly reported in NPC1 cases. HSM: hepatosplenomegaly, VM: visceromegaly, SM: splenomegaly. Blue: hepatomegaly; orange: splenomegaly; grey: visceromegaly; yellow: none; light blue: unknown

As expected, visceral symptoms were very frequent among the EIF NPC patients (Figure 2). The presence of visceromegaly has been noted in 80/89 cases (89.9%). A total of 54/89 were determined as hepatosplenomegaly (HSM), 11/89 as splenomegaly (SM), and 15/89 as visceromegaly (VM). In four cases, there was no visceromegaly and status of visceromegaly was unknown in five cases. Prolonged jaundice was reported in 30/89 cases.

### 2.3. Presentation and Progression of Neurological Symptoms

**Figure 3:**
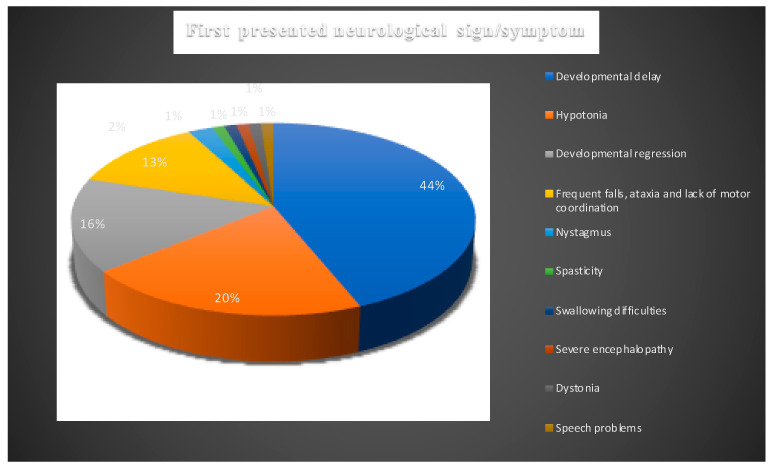
Presenting neurological signs and symptoms of all NPC1 patients. Blue: developmental delay; orange: hypotonia; silver: developmental regression; yellow: frequent falls, ataxia, and lack of motor coordination; light blue: nystagmus; green: spasticity; navy blue: swallowing difficulties; orange-red: severe encephalopathy; grey: dystonia; mustard: speech problems.

The time of onset and the type of neurological symptoms that patients experience predict the general disease course, progression, and life span in NPC. In Table 2 and Figure 3 we detailed the initial reported neurological symptoms and/or signs and the age of their onset.

In order to define the typical neurological features in a better categorized cohort of patients with EIF NPC, we removed patients with very limited information, i.e., those with “developmental delay” as the only clinical characteristic, from our calculations (Table 3).

This narrowed the cohort down to 43 patients with at least two neurological signs or symptoms. Developmental delay remained the most common first reported symptom which was noted in 31/43 (72%). Developmental delay was noticed in 17/43 of cases in the first year of life and 8/43 between 13 and 24 months of age. In six patients age of onset of the delay was not reported.

Hypotonia was the second most frequently reported initial symptom which was recorded in 13/43 patients. While it was reported in 11/43 patients in first 12 months of life, 1/43 patients presented with hypotonia between 13 and 24 months (Figure 3). In one case, the age onset of hypotonia was not identified. In 12 cases, hypotonia was noted to accompany developmental delay.

A total of 12/43 cases were reported to have exhibited developmental regression which means losing acquired functions or failing to progress after a normal developmental period, indicating a neurodegenerative disorder. Regression was described to occur in the first year of life in 3/43 patients, in the second year of life in 5/43 patients, and was not defined in 4/43.

Frequent falls, lack of motor coordination, and ataxia were reported as a presenting symptom in 10/43 patients; 3/10 in the first 12 months and 7/10 between 13 and 24 months. Frequent falls and ataxia were associated with developmental delay in 5/10 patients.

Spasticity, dystonia, and dysphagia were the other initial neurological symptoms described; each was reported in one patient occurring in the first 12 months of age.

Nystagmus was noted as a presenting symptom in one patient accompanied by severe encephalopathy with uncontrolled movements, ataxia, and tremor at the age of 24 months.

**Figure 4:**
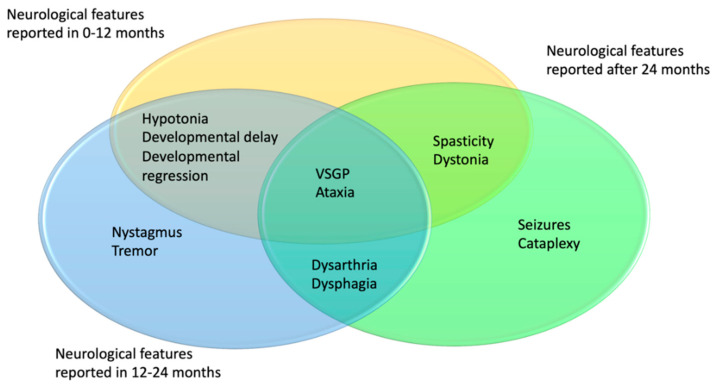
Distribution of age of onset for the neurological symptoms in NPC1 patients. In yellow 0–12 months, blue 13–24 months, green 24+ months.

Descriptions of these 43 patients had varying degrees of detail regarding the neurological progression of the disease. Age distribution of neurological symptoms and signs is shown in Figure 4. In 7/43 patients, additional symptoms were described in the first 24 months of life: VSGP in five cases, ataxia, dysphagia, and dysarthria in one case and one patient was described to display both ataxia and cataplexy. Most additional symptoms were described to have occurred after 24 months of age.

Vertical supranuclear gaze palsy and dysarthria were the most common neurological manifestations developed in the course of the disease; 16/43 (37.2%) and 13/43 (30.2%), respectively. VSGP was typically reported between 8 and 54 months.

A total of 10/43 cases developed cataplexy, which is a sudden and transient episode of muscle weakness and loss of consciousness lasting a few seconds and followed by full conscious awareness. The onset of cataplexy was described to have occurred between the ages of 2–5 years. A total of five of them were determined as gelastic cataplexy which is triggered by laughter.

Dysphagia and swallowing difficulties were reported in 9/43 patients between the ages of 13 and 60 months. A total of 8/43 patients developed seizures between the ages of 30 and 70 months. Ataxia/abnormal gait was described in 5/43 patients during the follow-up. A total of 4/5 patients with ataxia were also reported to have dysarthria. Dystonia was described in 5/43 cases and spasticity in 4/43.

### 2.4. Developmental Milestones

Delay in reaching normal developmental milestones is the major presenting feature of EIF NPC. Unfortunately, little detail was provided in the description of the milestone achievement and therefore it was not possible to tease out specific problems in most cases.

**Figure 5:**
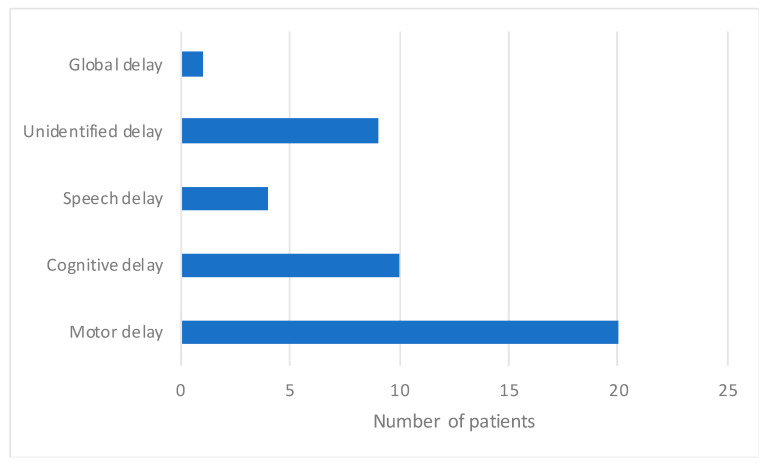
Distribution of reported developmental delay in NPC1 patients.

The distribution of description of developmental delay is shown in Figure 5. Developmental delay was reported in 31/43 patients. Motor development delay was the most commonly mentioned abnormality among the EIF patients, reported in 20/43 patients. In 10/43 patients motor delay was accompanied by cognitive delay and in 3/43 patients by speech delay. In 1/43 case only speech delay was mentioned. Unspecified developmental delay was mentioned in 9/43 patients and global development delay was noted in 1/43 patients. Hearing and visual impairment were reported in one patient.

### 2.5. Age and Cause of Death

Age of death was reported in 31/89 patients. Median age of death was 48 months (range 7–132 months). Cause of death was only reported in 7/31 cases and all of them were due to respiratory failure. A total of 3/31 of these patients received miglustat treatment. A total of two of them started miglustat therapy at the age of 7 years, however they only received treatment for 1 week. One other patient started miglustat therapy at the age of 20 months and after 13 months of therapy, the patient was deceased due to the respiratory complications.

### 2.6. Neuroimaging Findings

Neuroimaging data was available in 10/81 patients and only one of them was reported as normal [51]. Cerebral atrophy was mentioned in nine patients [42,46,49,50]. Deep white matter signal abnormalities were seen in six patients [42,49].

### 2.7. Additional Diagnostic Investigations

The filipin test, based on the demonstration of accumulated unesterified cholesterol within the lysosomes of cultured fibroblasts, for many years was regarded as the gold standard for NPC diagnosis, however, more recently it has been used less frequently compared with analysis of other biomarkers [12]. As expected, most of the historical patients underwent filipin staining. Filipin test was used to confirm the diagnosis in patients in whom only a single *NPC1* mutation was identified. The other tests that were commonly employed to assist in the diagnostic process in the past included bone marrow aspiration and chitotriosidase activity assay. These analyses are not recommended anymore as they are not as sensitive or specific as the newer tests such as analyses of oxysterols, NPC bile acids, and lyso-SM-509 levels [3].

### 2.8. Genotype Phenotype Correlation

In order to identify *NPC1* mutations that confer EIF phenotype we selected 42 patients with at least two neurological signs or symptoms from Table 2. As above, patients who presented with only mild developmental delay were not included in order to avoid potential inclusion error (Table 2). We were able to identify mutations that in homozygous state confer EIF phenotype (Table 3). Some of these mutations were also seen in homozygous state in LIF phenotype suggesting a degree of overlap between the two groups. A combination of the severe (EIF causing) mutation with a milder one was seen to confer the later onset disease.

A total of 15 mutations occurred in homozygous state in EIF patients (Table 4). They included two gross deletions, one small deletion, two insertions and deletions that result in a frame shift, two splice site mutations, and eight missense mutations.

A total of 9/15 mutations: deletion of exon 1+promoter, c.3578_3591 + 9del, c.385delT, p.C63fsX75, IVS21-2delATGC, c. 2740T>A (p.C914S), c.3584G>T (p.G1195V), c.3478-6T>A, c.960_961dup (p.A321Gfs*16) were only reported in EIF patients.

c.3503G>A (p.C1168Y) was identified in one EIF patient but also in two LIF patients in a homozygous state [37,55]

c.1415T>C (p.L472P) in exon 9 of the *NPC1* gene was found in two Iranian patients with EIF [53]. It was previously reported in two other Iranian patients in a homozygous state; one of them was also EIF patient, while the other was a LIF patient [45].

c.3394G>C (p.A1132P), a missense point mutation in exon 22, was reported in a Greek patient with an EIF. The same report described a LIF patient who died at the age of 14 years with the same mutation [13].

Homozygous gross deletion of exon 1+promoter was reported in one patient [48]. In previous reports, three patients were also described with a EIF phenotype even though they had different gross deletions of the NPC1 gene in a compound heterozygous state [39,57].

c.1211G>A (p.R404Q) was found in Bedouin-Israeli patients in homozygous state who had EIF, LIF, and EISL phenotypes [47]. It was also detected in a French EIF patient in a compound heterozygous state with c.709C>T (p.237S) [56]. A total of two juvenile patients were reported to have c.1211G>A (p.R404Q); one as the only mutation, the other was in combination with c.1133T>C (p.Vl378A) [37].

c.3104C>T (p.A1035V) was reported in two EIF patients; one in homozygous state, the other was reported in combination with IVS23+1G>A [52]. It was also reported in a juvenile patient with an unknown second allele [52]. Another juvenile patient had c.3104C>T (p.A1035V) in combination with c.3019C>G (p.P1007A), again suggesting that this latter mutation confers milder phenotype [58].

Besides occurring in homozygous state, there were two patients referred in literature being compound heterozygotes for IVS23 +1G > A (c.3591+1G>A) with c.3104C>T (p.A1035V) or c.3182T>C (p.I1061T), respectively, who displayed the EIF phenotype and another patient who had this mutation in combination with c.2090C>T (p.V697A) and showed the LIF phenotype, confirming the hypothesis that this mutation confers more severe illness [36,52,59].

c.3107C>T (p.T1036M) is a severe mutation which was associated with EIF phenotype in a homozygous state [42]. It was reported in two other EIF patients in combination with c.3557G>A (p.R1168H) [37]. c.3107C>T (p.T1036M) was found in two LIF and one juvenile patient, in combination with c.3182C>T (p.I1061T) and c.2861C>T (p.S954L) mutations that typically confer later onset phenotypes [37].

c.3557G>A (p.R1186H) mutation was found in the EIF phenotype when it occurs in combination with c.3107C>T (p.T1036M), c.826T>C (p.Y276H), c.3614C>A (p.T1205K), and c.2196dupT (p.P733Sfs*9) [36,37]. It was also reported in LIF patients in combination with c.1421C>T (p.P474L), c.3019C>G (p.P1007A), c.826T>C (p.Y276H), c.3182T>C (p.I1061T), c.2830G>A (p.D944N), and c.2196dupT (p.P733Sfs*9) [36,60]. c.3557G>A (p.R1186H) mutation was also detected in juvenile patients when it occurs in combination with a c.2861C>T (p.S954L) mutation known to confirm juvenile phenotype [36,60]. c.3557G>A (p.R1186H) was found in a LIF patient and a patient with isolated splenomegaly without neurological presentation in a homozygous state, which suggests that the severity of this mutation depends on the additional genomic factors [36].

While c.1553G>A (p.R518Q) was found in two EIF patients in combination with c.2795dupA, it was detected in a LIF patients in a homozygous state and in combination with c.1172A>G (p. E391G) [61,62]. It was shown in juvenile patients in combination with c.3019C>G (p.P1007A) and c.494C>T (p.A165V) [37,61].

While c.3614C>G (p.T1205R) was reported in an EIF patient with c.3614C>A (p.T1205K), it was also noted in a LIF patient in a combination with c.1553G>A (p.R518Q) suggesting that the latter mutation confers milder phenotype [63]. It was detected in a patient presented as an EISL phenotype in a compound heterozygous state with c.1261C>T (p.Q421X) [36].

### 2.9. Early Infantile Neurological Onset NPC Due to NPC2 Mutations

Analysis of massively parallel sequencing data sets revealed that incidence rate for *NPC2* mutations is extremely rare at 1/2,858,998 [16]. Therefore, only a few small case series were reported [64,65,66,67,68].

An observational, retrospective study of NPC patients from Czech Republic revealed 56 cases and only one of them had *NPC2* genotype with an EIF phenotype. This female patient presented with pulmonary involvement, psychomotor retardation, central hypotonia, and moderate hepatosplenomegaly at the age of 1 year. Spasticity and cognitive deterioration occurred during the second year of life and the patient died at 4 years of age due to respiratory insufficiency [36].

Retrospective data for UK-based patients with NPC revealed two cases with *NPC2* mutations among a total of 112 patients. Both of them were LIF patients with few insidious neurological manifestations [37].

A collaborative multicenter study from Italy which aimed to characterize the molecular basis of NPC reported eight *NPC2* patients, six of whom presented with severe phenotypes including EISL and EIF; two patients had an adult phenotype [41].

The clinical, biochemical, and molecular findings of 14 NPC cases diagnosed in Greece were demonstrated and only 1/14 had *NPC2* mutations causing a LIF [54].

A prospective epidemiologic cohort study from Turkey aimed to investigate the frequency of NPC mutations in consanguineous families with at least one homozygous family member. A total of 3/4 randomly selected probands had *NPC2* mutations, which is likely to demonstrate a founder effect in this region [67].

According to the review of literature, 16 *NPC2* cases were found with EIF (Table 5). Cases which did not have neurological symptoms were excluded. A total of 8/16 cases were female, 3/16 were male, and 5/16 were unknown gender.

While visceral symptoms were found in all patients, visceromegaly was noted in 15/16 patients (93.8%); 9/16 had HSM, 4/16 were SM, and 2/16 had HM. Pulmonary involvement was reported in 12/16 patients. Prolonged jaundice was noted in 7/16 patients.

Age of neurological onset was reported in 14 patients with median age of onset 7.5 months (range 0–24). Hypotonia and developmental delay were the most common initial neurological symptoms, reported in 10/16 and 8/16 cases, respectively. While hypotonia presented in 8/16 patients in the first 12 months, it was reported to have occurred between 12 and 24 months in 2/16 patients. Similar to that developmental delay was noted in 6/16 patients in the first year of life and 2/16 cases in the second year of life. In one case, abnormal gait accompanied hypotonia as a presenting neurological symptom.

**Figure 6:**
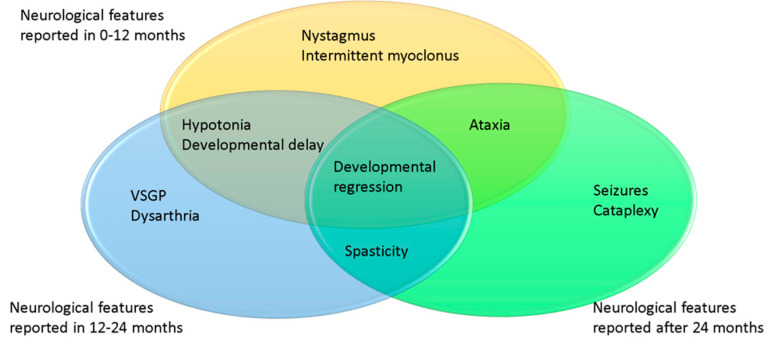
Age of onset of presentation for neurological symptoms in NPC2 patients. In yellow 0–12 months, blue 12–24 months, green 24+ months.

There was not enough data to detail the neurological progression and developmental milestones in NPC2 patients, mainly because most succumb in infancy. Distribution of neurological symptoms are shown in Figure 6. A total of three patients exhibited hypotonia with developmental delay during the follow-up. Dysarthria was reported in two cases. While VSGP was mentioned in one case, another patient developed mild horizontal saccade abnormalities and end-point nystagmus as well as intermittent myoclonus. Ataxia and cataplexy were reported in the same patient. One patient was reported to develop intellectual regression and another developed seizures at the age of 7 years.

A total of nine patients were reported as deceased with the median age of 10 months (range 4.5–48). In all nine patients, cause of death was reported as respiratory failure.

*NPC2* mutations causing EIF are shown in Table 6, based on mutation–phenotype association. c.58G>T (p.E20X) nonsense mutation was the most common reported in five cases in a homozygous state. c.58G>T creates a premature stop codon downstream of the signal peptide. All patients had early onset pulmonary involvement with neurological deterioration [36,40,64]. However, two other patients homozygous for the same mutation presented with hepatosplenomegaly without reported neurological symptoms, significant respiratory disease, and death at the age of 6 months [64]. It was also associated with respiratory failure and premature death without obvious neurological involvement in a French patient in combination with c.27delG (p.Leu10Serfs*25) [64]. It was also demonstrated in a LIF patient in a homozygous state [37].

c.352G>T (p.E118X) was the second most frequent mutation observed in four patients in a homozygous state. This nonsense mutation in exon 3 of *NPC2* leads to a premature stop codon and was associated with a severe clinical progression and death in the first 2 years of life [65,67]. It was reported in a German patient presented with visceromegaly, respiratory involvement, and early death without assigned neurological symptoms [64].

c.434T>A (p.V145E) was exclusively reported in two patients with EIF in a homozygous state.

The following mutations: c.436C>T (p.Q146X), c.199T>C (p.S67P), c.133C>T (p.Q45X), c.141C>A (p.C47X), and c.82+2T>C (IVS1+2T>C) were all reported in individual cases. All patients had severe pulmonary disease accompanied by neurological involvement. Homozygous c.436C>T (p.Q146X) was also reported in an Algerian patient with cholestatic icterus and hepatosplenomegaly with ascites [66,72]. c.82+2T>C (IVS1+2T>C) was also demonstrated in a Sri-Lankan patient in whom hepatosplenomegaly, severe pulmonary involvement with hypoxia, and severe nutritional problems were first detected aged 4.5 months without obvious neurological involvement [66].

c.434T>A (p.V145E), c.199T>C (p.S67P), c.133C>T (p.Q45X), c.141C>A (p.C47X) mutations were only associated with EIF.

One Chinese patient was found to be compound heterozygous for c.3G>C (p.M1I) and c.190+5G>A (IVS2+5G>A) mutations [46]. While c.3G>C (p.M1I) was only reported in this patient, c.190+5G>A (IVS2+5G>A) was reported in two juvenile cases in a homozygous state [54,64].

## 3. Discussion

This review intended to consolidate the knowledge of the EIF NPC clinical presentation in order to provide an up-to-date evaluation of the characteristics of this form of the disease. Granular understanding of the clinical symptom progression is of paramount importance to good clinical practice, accurate family counselling, and clinical trial design. A major limitation of this review based on literature search was the considerable heterogeneity among the included studies, particularly the way patient demographic and clinical characteristics were reported. Moreover, neurological data including the chronology of signs and symptoms and history of developmental progression were very limited. Therefore, in order to make the most reliable and accurate conclusions, we restricted the patient cohort and selected the cases with more detailed information in a consistent way. Several exclusion criteria were set to minimize the possibility of bias. Hence, clinical characteristics including the initial presentation, neurological and developmental progression, and molecular features of both EIF NPC1 and NPC2 group were set.

As expected, a remarkable proportion of EIF patients had a history of visceromegaly (80/89). However, only 30/89 patients had prolonged neonatal jaundice reported [1,36,37,38,46]. The presence of these symptoms and early referral to the treatment center is likely to allow early diagnosis of NPC, provided appropriate diagnostic measures are instigated [12,30,73].

Lung involvement is a prominent feature of the NPC2 disease. It is thought to be caused by the lung infiltration by foamy macrophages and is associated with pulmonary alveolar proteinosis, unlike NPC1 patients in whom respiratory complications are typically due to recurrent aspirations and infections [47,74,75,76,77,78,79,80].

Failure to progress along the expected developmental milestones is usually the first opportunity to identify the neurological abnormality in a patient with EIF NPC [2]. Onset of neurological symptoms predicts the disease progression and the expected lifespan [1,12]. In the EIF group with two or more neurological symptoms the median age of onset was 12 months (range 0–24) in the NPC1 and 7.5 months (range 0–24) in the NPC2. The most frequent neurological symptoms were developmental delay and hypotonia in both groups. NPC1 patients also displayed developmental regression, “frequent falls and ataxia”, spasticity, dysphagia, and nystagmus as an initial neurological symptom. Both hypotonia and developmental delay occurred in the first year of life more often than in the second 12 months.

From the reviewed data it was difficult to obtain granularity in the developmental delay data (Figure 5). Whilst this symptom is crucial for early diagnosis of EIF, it is well known that cases with LIF and even juvenile types of the disease may display some developmental delay. Hence, in this review we tried to select patients with features that provided more reassurance regarding the specific NPC form. Nevertheless, it is difficult to be absolutely confident in this selection. Furthermore, as an isolated symptom developmental delay leads to a large list of differential diagnoses and hence may not be helpful in the diagnostic process.

The age of presentation of various neurological symptoms is shown in Figure 4 and Figure 6. VSGP and dysarthria were the most common followed by cataplexy, dysphagia, seizures, ataxia, and spasticity. Gelastic cataplexy and VSGP have been reported as highly specific for NPC and in particular VSGP is often suggested as the initial neurological sign. Hence, we were keen to explore whether this statement holds true for the EIF [30,73,81]. In the cohort with at least two neurological signs or symptoms, we found that VSGP was reported in 16/43 and mostly developed earlier (median 30 months, range 8–54 months) than cataplexy, which was reported in 10/43 cases (median 36, range 24–60 months). Thus, ophthalmological assessment is an important part of the follow-up management but absence of VSGP as a presenting feature cannot exclude EIF type of NPC.

Brain imaging in NPC is usually nonspecific early in the disease and is often reported as cerebellar and cerebral atrophy at later stages of progression. In the EIF cohort reviewed here we found that brain atrophy and white matter signal abnormalities were the most frequent changes reported [42,49,50,51] consistent with other studies [82].

We have not specifically interrogated the data on laboratory investigations performed in this cohort. Typically filipin staining and other biomarker analyses such as levels of oxysterols, lysoSM509 ratio, NPC specific bile acids are used for diagnostic screening, whilst molecular tests provide the definitive result in most, although not in all, cases [3,15,17].

According to the literature, the EIF patients live shorter after the onset of neurological disease than those patients with later presentations [1,18,20,37,83]. Median age of death was earlier in the NPC2 (median 10 months, range 4.5–48) compared to NPC1 patients (median 48 months, range 7–132). In agreement with individual reports, respiratory complications were the most frequent cause of death in EIF patients in both NPC1 and NPC2 groups. The earlier death may be due to the more severe pulmonary involvement in NPC2 cases. [64,66,67,70]. NPC1 patients usually die following the neurological disease progression, leading to respiratory complications such as aspiration pneumonia and respiratory failure [84].

We interrogated professional version of the Human Gene Mutation Database (www.hgmd.cf.ac.uk) as an up-to-date source of disease mutations in order to verify our findings from the literature review. It lists a total of 524 *NPC1* defects that include 344 missense and nonsense mutations, 73 small deletions, 47 small insertions, 43 mutations affecting splicing, 12 gross deletions, three small indels, and two gross insertions/duplications. The *NPC1* gene encodes NPC1 protein has 13 transmembrane domains, a sterol-sensing domain, a cysteine rich luminal loop, and a highly conserved domain with a leucine-zipper motif in the N-terminal tail. Most common mutations associated with later phenotypes including p.I1061T, p.P1007A, and p.G992W result in changes in the cysteine-rich loop which has an important role in the cholesterol transport chain [85].

The following mutations so far have only been reported to only cause EIF in homozygous state: gross deletion of exon 1+promoter, c.3578_3591 + 9del, c.385delT, p.C63fsX75, IVS21-2delATGC, c. 2740T>A (p.C914S), c.3584G>T (p.G1195V), c.3478-6T>A, c.960_961dup (p.A321Gfs*16) (Table 3). IVS23+1G >A is predicted to be a severe mutation, which causes EIF even in combination with p.A1035V or p.I1061T. c.1415T>C (p.L472P) and c.3394G>C (p.A1132P) mutations were detected in both early and LIF patients in homozygous state. c.3107C>T (p.T1036M) and c.1553G>A (p.R518Q) often cause EIF, however, when in combination with late onset associated mutations such as c.3182C>T (p.I1061T), c.3019C>G (p.P1007A), and c.2861C>T (p.S954L), they mostly present with a later onset phenotypes.

The *NPC2* gene encodes a small lysosomal/late-endosomal glycoprotein that is ubiquitously expressed and plays a role in cholesterol trafficking [8,9,86]. Twenty-eight disease causing mutations have been reported so far (www.hgmd.cf.ac.uk) including 19 missense/nonsense mutations, four mutations affecting splicing, three small deletions, one small insertion/deletion, and one gross deletion. c.58G>T (p.E20X) and c.352G>T (p.E118X) were the most common mutations associated with EIF, however they were also reported in non-neurological phenotype with significant pulmonary involvement and visceromegaly. Whilst the patients did not appear to show obvious neurological features, it is likely that the early death pre-empted symptom evolution. c.434T>A (p.V145E), c.436C>T (p.Q146X), c.199T>C (p.S67P), c.133C>T (p.Q45X), c.141C>A (p.C47X), and c.82+2T>C (IVS1+2T>C) mutations were also reported in EIF patients in a homozygous state. c.434T>A (p.V145E), c.199T>C (p.S67P), c.133C>T (p.Q45X), c.141C>A (p.C47X) mutations were only associated with EIF phenotype (Table 5). There was only one patient with compound heterozygous c.3G>C (p.M1I) and c.190+5G>A (IVS2+5G>A) mutations. c.190+5G>A (IVS2+5G>A) was reported in a juvenile phenotype associated mutation [54,64]. c.3G>C (p.M1I) is predicted to be a severe mutation which led to an EIF even in combination with a juvenile phenotype associated mutation.

There is a dearth of effective treatments available for NPC. Management is mainly symptomatic to increase the quality of life [1,12]. Patients with dysphagia should be closely monitored to avoid serious lung infections secondary to aspiration and to ensure adequate nutrition. Some patients need gastrostomy to maintain daily caloric intake [18,84]. Antiepileptic drugs are used although typically seizures are difficult to control [17]. Although tricyclic antidepressants have been used historically for cataplexy, their effect is very limited [17]. Dystonia and tremors may respond to anticholinergic drugs at least transiently, in some patients. Other drugs used for tone management include trihexyphenydil, benzodiazepines, botulinum toxin, and gamma-aminobutyric acid [17]. Physiotherapy can be used to delay the onset of contractures.

Miglustat is the only disease specific therapy approved for treatment of NPC worldwide. Miglustat is an iminosugar that inhibits glycosphingolipid synthesis. It has been reported to slow neurological deterioration and improve survival in different NPC cohorts [42,84,87,88]. Recently, a large retrospective observational study that used a multinational registry and five large national cohorts suggested that miglustat effect was statistically significant in improving the survival of LIF and juvenile patients, but not those in EIF group [89]. We could not obtain sufficient data on the miglustat use in the EIF group reviewed here to comment on its effect.

A number of experimental therapy trials are currently at different stages of clinical development. Arimoclomol upregulates molecular chaperones in cells including Hsp70, which increases the expression of mutant protein forms that may retain some of the function. The initial results of an Orphazyme-sponsored prospective, randomized, double-blind, placebo-controlled study suggested potential efficacy (NCT02612129) [90]. Hydroxypropyl-beta-cyclodextrin (HPβCD) is a molecule, which was shown to reduce neuronal cholesterol and ganglioside storage, decrease Purkinje cell death, and increase lifespan in many animal model studies [91,92,93,94]. A phase I/IIa trial of intrathecal HPβCD demonstrated a slower neurological disease progression in treated patients (NCT02534844, NCT01747135). The clinical trials investigating intravenous (sponsored by CTD Holdings) and intrathecal (sponsored by Malinckrodt) HPβCD, as well as the combined administration (see clinicaltrials.govc) are ongoing [95,96]. Acetyl-DL-leucine, an acetylated derivative of a natural amino acid, significantly improved ataxic symptoms without side effects and quality of life [97,98]. A phase 2 clinical trial investigating the efficacy of Acetyl-L-Leucine sponsored by IntraBio is currently in progress (NCT03759639). Unfortunately, most of the clinical trials initiated so far excluded patients with early forms of the disease.

There has been a suggestion that bone marrow transplantation (BMT) may be effective in NPC2 as NPC2 is a soluble and secreted protein. However, only one NPC2 case treated with BMT and a long-term follow up could be found in the literature. A LIF NPC2 patient, presented with HSM and respiratory symptoms from the neonatal period including tachypnoea, recurrent infections, and oxygen dependence underwent BMT at the age of 16 months. Gradual improvement in respiratory symptoms and hepatosplenomegaly was reported by the age of 3 years. The child started to walk independently at the age of 24 months. However, after the age of 2 years the patient started regressing and at 33 months he no longer had any words. At 46 months of age, there was a decrease in his socialization with a reduction in vocalization. At the age of 63 months seizures started and became gradually intractable [99,100].

Gene therapy is a rapidly evolving field and a number of pre-clinical studies have shown that it may hold potential in treating NPC [101,102,103]. These studies have involved adeno-associated viral vectors to deliver a functional copy of the *NPC1* gene to a mouse model of NPC and reported neurological and systemic improvements and increased survival.

## 4. Materials and Methods

This review was based on a search through MEDLINE database using PubMed as the search engine. All relevant articles, original articles, case reports, and reviews published through April 2020 were included. The database was searched using the following medical subject headings (MeSH terms) “Niemann Pick Disease type C” and “Niemann Pick Disease”. Furthermore, the references of the articles were investigated by hand for related articles. Our main focus were patients with EIF phenotype, therefore only cases with neurological symptoms in the first 2 years of life were included. Cases with late infantile (LIF), juvenile, and adult phenotypes were excluded. Cases presented with acute liver failure and treated with liver transplantation were not included in order to ensure uniformity. To evaluate the characteristics of EIF neurological disease, cases with “developmental delay” reported as the only symptom were also excluded. Only cases with at least one identified mutation in *NPC1* or *NPC2* were selected. In cases where only one mutation was identified, the diagnosis had to be supported by a biomarker test such as filipin staining of cultured skin fibroblasts. Other exclusion criteria used were (1) the article does not have abstract or the abstract is not available from the included electronic database; (2) the full text of the article is not available in English.

## 5. Conclusions

These data derived from an extensive review of NPC literature focused on the EIF type of NPC and provides an overview of the disease, with particular emphasis on neurological course and genetic features. Identification of mutations determining the EIF will help predict the disease progression and structure future clinical trials of novel therapies. Inevitably, only prospective natural history studies with set inclusion and exclusion criteria as well as detailed assessment categories could provide the granularity for the time course of the disease. This can only be achieved by close interaction of many international centers and family organizations.

## Figures and Tables

**Table 1:** Summary of the Niemann Pick disease type C (NPC) studies.

Reference	Country	Methodology	Total Number of Patients NPC1/NPC2	Number of EIF Patients NPC1/NPC2	Most Common Neurological Symptom among EIF Patients	Median Age of Death (Months)
[36]	Czech Republic	Observational retrospective	55/1	6/1	Psychomotor Retardation/regression	60
[37]	UK	Observational retrospective	110/2	8/0	DD, ataxia, dysarthria	65
[2]	International	Observational prospective	134/3	16	DD, dysphagia and VSGP	
[38]	Spain	Mutation screening	40/0	12		
[39]	Spain	Mutation screening	30/0	10		
[40]	Italy	Mutation screening	32/2	11/2	Hypotonia, ataxia	
[41]	Italy	Mutation screening	97/8	21/3		
[42]	France	Prospective open-label	19/1	8/0	Hypotonia, DD	
[43]	Egypt	Observational descriptive	23/0	6/0		
[44]	Iran	Observational descriptive	21/0	3/0	DD	
[45]	Iran	Observational case series	11/0	5/0		
[46]	China	Biochemical/genetic screening	11/1	6/1	Frequent falls, DD	
[21]	Germany Switzerland	Cross-sectional analysis	41/1	5/1		
[47]	Israel	Descriptive	12/0	5/0		

**Table 2:** Demographic, clinical, and molecular features of early infantile neurological onset disease with *NPC1* mutations.

Patient No	Initial Neurological Symptom/s (Age First Reported in Months)	Visceral Symptoms	Sex	Ethnicity	Allele 1	Allele 2	Age of Death (Months)	Ref.
1	Swallowing difficulties lack of motor coordination (3)	HSM, PJ	F	Spanish	del promotor+exon1	del promotor+exon1	NK	[48]
2	Severe encephalopathy with uncontrolled movements, ataxia, tremor, nystagmus (24)	SM	M	Spanish	c.385delT	c.385delT	NK	[48]
3	NK	HSM, PJ	F	Spanish	c.2830G>A (p.D944N)	c.3104C>T (p.A1035V)	NK	[48]
4	NK (22)	HSM	M	Spanish	c.1757delA	c.2746_2748delAAT	NK	[48]
5	Hypotonia (5)	HSM, PJ	F	French	c.1138C>T (p.L380F)	c.2872C>T (p.R958X)	NK	[42]
6	Hypotonia, DD (6)	HSM, PJ	F	French	p.C63fsX75	p.C63fsX75	NK	[42]
7	Hypotonia, DD (7)	HSM	F	French	c.3614C>G (p.T1205R)	c.3614C>A (p.T1205K)	NK	[42]
8	Hypotonia, DD (9)	HSM, PJ	F	French	IVS21-2del ATGC	IVS21-2del ATGC	33	[42]
9	Hypotonia, DD (9)	HSM, PJ	F	French	c.3584G>T (p.G1195V)	c.3584G>T (p.G1195V)	NK	[42]
10	Hypotonia, DD (10)	HSM, PJ	F	French	c. 1298C>T (p.P433L)	IVS14+1G>A	NK	[42]
11	Hypotonia, DD (12)	HSM, PJ	F	French	c. 1298C>T (p.P433L)	p.T1205fs	NK	[42]
12	Hypotonia, DD (12)	HSM, PJ	F	French	c.3107C>T (p.T1036M)	c.3107C>T (p.T1036M)	NK	[42]
13	Frequent falls, DD (12)	SM	F	Chinese	c.1501G>T (p.D501Y)	c.1800delC (P.I601FfsX13)	NK	[46]
14	Frequent falls, DD (18)	SM	M	Chinese	c.416dupC (p.N140KfsX30)	c.1832A>G (p.D611G)	NK	[46]
15	Frequent falls, DD (12)	HSM, PJ	M	Chinese	c.2177G>C (p.R726T)	c.3734_3735delCT (p.P1245RfsX12)	NK	[46]
16	DD (20)	HSM	M	Chinese	c.2230_2231delGT (p.V744SfsX27)	c.3734_3735delCT (p.P1245RfsX12)	NK	[46]
17	Frequent falls (15)	SM	F	Chinese	c.1553G>A (p.R518Q)	c.2795dupA	NK	[46]
18	Frequent falls (20)	SM	M	Chinese	c.1553G>A (p.R518Q)	c.2795dupA	NK	[46]
19	DD (NK)	No	M	UK	c.2819C>T (p.S940L)	NK	92	[37]
20	DD (NK)	VM, PJ	F	UK	c.3557G>A (p.R1186H)	c.3107C>T (p.T1036M)	101	[37]
21	DD (NK)	VM, PJ	F	UK	c.3557G>A (p.R1186H)	c.3107C>T (p.T1036M)	85	[37]
22	DD (17)	No	F	UK	c.3503G>A (p.C1168Y)	c.3503G>A (p.C1168Y)	77	[37]
23	DD (NK)	PJ, VM	F	UK	c.3578_3591 + 9del	c.3578_3591 + 9del	40	[37]
24	DD (NK)	PJ, VM	F	UK	c.2801G>A (p.R934Q)	c.2978del (p.G993EfsX4)	53	[37]
25	DD (12)	HSM, PJ	M	Czech	c.3557G>A (p.R1186H)	NK	60	[36]
26	DD (12)	HSM	F	Czech	c.3182T>C (p.I1061T)	c.3591+1G>A	132	[36]
27	DR (20)	HSM	F	Czech	c.1812dupT (p.Ala605Cysfs*2)	c.3558delC (p.A1187Rfs*54)	48	[36]
28	Speech retardation (22)	HSM, PJ	F	Czech	c.3557G>A (p.R1186H)	c.826T>C (p.Y276H)	NK	[36]
29	DR, ataxia (18)	HSM	F	Czech	c.3557G>A (p.R1186H)	c.2196dupT (p.Pro733Serfs*10)	NK	[36]
30	DD, ataxia (22)	SM	F	Czech	c.3557G>A (p.R1186H)	c.3614C>A (p.T1205K)	72	[36]
31	DD (12)	HSM	F	Spanish	c.319delc	Nucleotide +5 at intron 18	NK	[49]
32	Speech regression (18)	SM	F	Japanese	c.2108T>C (p.F703S)	c.2438C>G (p.S813X)	108	[50]
33	Hypotonia (2), DD (4)	HSM, PJ	M	Japanese	c.2783A>C (p.Q928P)	c.3008T>G (p.L1003R)	NK	[51]
34	Hypotonia (1)	HSM	NK	Spanish	c.2826G>T (p.W942C)	c.2883_2897del15 (p.Ile962_Phe966del)	NK	[38]
35	Hypotonia, DD (12)	HSM	NK	Spanish	c.3104C>T (p.A1035V)	c.3104C>T (p.A1035V)	NK	[38]
36	Hypotonia (Newborn)	HSM	NK	Spanish	c.530G>A (p.C177Y)	c.2876T>A (p.V959E)	NK	[38]
37	Motor regression, spastic tetraparesis (12)	No	NK	Spanish	c.955+1G>A (IVS7+5G>A)	c.2826G>T (p.W942C)	NK	[38]
38	Nystagmus (8)	HSM	NK	Spanish	c.1935T>A (p.C645X)	c.3236T>C (p.F1079S)	NK	[38]
39	DD (3)	HSM, PJ	M	Iranian	c.2925_2928delCTGC (p.C976fs)	c.2925_2928delCTGC (p.C976fs)	NK	[44]
40	NK (8)	VM	F	Egyptian	c.3380dupT (p.M1127Ilfs*131)	c.3380dupT (p.M1127Ifs*131)	NK	[43]
41	NK (4)	VM	M	Egyptian	c.425_426delAA (p.K142Rfs*27)	c.425_426delAA (p.K142Rfs*27)	NK	[43]
42	NK (6)	VM	M	Egyptian	c.2872C>T (p.R958X)	c.2872C>T (p.R958X)	NK	[43]
43	NK (4)	VM	M	Egyptian	c.2245+1G>A	c.2245+1G>A	NK	[43]
44	NK (9)	VM	M	Egyptian	c.2972_2973delAG (p.Q991Rfs)	c.2972_2973delAG (p.Q991Rfs)	NK	[43]
45	NK (10)	VM	F	Egyptian	c.2972_2973delAG (p.Q991Rfs)	c.2972_2973delAG (p.Q991Rfs)	NK	[43]
46	NK (4)	VM	M	Egyptian	Duplication/multiple copies of exons 10 and 11	Duplication/multiple copies of exons 10 and 11	NK	[43]
47	NK (8)	VM	F	Egyptian	c.3032_3038delins10bp (p.C1011*)	c.3032_3038delins10bp (p.C1011*)	NK	[43]
48	NK (18)	VM	F	Egyptian	c.2972_2973delAG (p.Q991Rfs)	c.2972_2973delAG (p.Q991Rfs)	NK	[43]
49	Hypotonia (24)	HSM	F	Portuguese	IVS23+1G>A	IVS23+1G>A	NK	[52]
50	DD (12)	HSM	NK	Portuguese	c.3104C>T (p.A1035V)	c.3104C>T (p.A1035V)	54	[52]
51	Hypotonia (23)	HSM, PJ	NK	Portuguese	IVS23+1G>A	c.3104C>T (p.A1035V)	36	[52]
52	NK (18)	VM	M	Egyptian	c.2872C>T (p.R958X)	c.2872C>T (p.R958X)	NK	[43]
53	NK (11)	VM	M	Egyptian	c.451_452delAG (p.S151Ffs*18)	c.451_452delAG (p.S151Ffs*18)	NK	[43]
54	DR (NK)	HSM	F	Iranian	c.2920_2923delCCTG (p.C976Ffs*6)	c.2920_2923delCCTG (p.C976Ffs*6)	24	[45]
55	DR (NK)	HSM	M	Iranian	c. 2740T>A (p.C914S)	c. 2740T>A (p.C914S)	NK	[45]
56	DR (NK)	HSM	F	Iranian	c.1415T>C (p.L472P)	c.1415T>C (p.L472P)	NK	[45]
57	DR (NK)	HSM	F	Iranian	c.3478-6T>A	c.3478-6T>A	28	[45]
58	DR (NK)	HSM	F	Iranian	c.960_961dup (p.A321Gfs*16)	c.960_961dup (p.A321Gfs*16)	7	[45]
59	DD, Ataxia (24)	HSM	F	Greek	c.3394G>A (p.A1132P)	c.3394G>A (p.A1132P)	NK	[13]
60	NK	HSM	NK	Italian	p.F284LfsX26	p.F284LfsX26	NK	[40]
61	NK	SM	NK	Italian	c.2972_2973delAG (p.Q991Rfs)	c.2972_2973delAG (p.Q991Rfs)	NK	[40]
62	NK	HSM	NK	Italian	c.1819C>T (p.R607X)	c.3614C>A (p.T1205K)	36	[40]
63	NK	SM	NK	Italian	c.93_94delTG (p.C31WfsX26)	c.93_94delTG (p.C31WfsX26)	NK	[40]
64	NK	HSM	NK	Italian	c.464-2A>C	c.464-2A>C	NK	[40]
65	NK	HSM	NK	Italian	c.464-2A>C	c.464-2A>C	NK	[40]
66	NK	HSM	NK	Italian	c.3467A>G (p.N1156S)	c.3467A>G (p.N1156S)	NK	[40]
67	NK	HSM	NK	Italian	c.3613dupA (p.T1205NfsX53)	c.3613dupA (p.T1205NfsX53)	NK	[40]
68	NK	HSM	NK	Italian	c.2800C>T (p.R934X)	c.2872C>T (p.R958X)	36	[40]
69	NK	HSM	NK	Italian	c.2829C>G (p.I943M)	NK	NK	[40]
70	NK	NK	F	German	c.2071C>T (p.P691S)	c.2279_2281TCTdel (p.Phe760del)	NK	[21]
71	NK (12)	NK	F	German	c.352_353delAG p.(Gln119Valfs*8)	c.352_353delAG p.(Gln119Valfs*8)	72	[21]
72	NK (24)	NK	F	German	c.3047A>T (p.H1016L)	c.3182T>C (p.I1061T)	NK	[21]
73	NK (24)	NK	F	German	p.S940L	NK	NK	[21]
74	NK (24)	NK	M	German	c.3019C>G (p.P1007A)	c.2873G>A (p.R958Q)	NK	[21]
75	DD (5)	SM, PJ	M	Iranian	c.1415T>C (p.L472P)	c.1415T>C (p.L472P)	NK	[53]
76	DD, DR (9)	SM	F	Iranian	c.1415T>C (p.L472P)	c.1415T>C (p.L472P)	NK	[53]
77	Hypotonia, DD (NK)	PJ	M	Greek	IVS23 + 3insT (c.3591 + 3insT)	NK	42	[54]
78	Hypotonia, DD, Dystonia (5)	HSM, PJ	F	Greek	c.852delT (p.F284Lfs*26)	del promotor+exon1-10	26	[54]
79	Hypotonia (3)	PJ, HSM	M	Greek	c.275A>G (p.Q92R)	c.3557T>C (p.C119*)	NK	[54]
80	DD (9)	PJ, HSM	M	Greek	c.3265G>A (p.E1089K)	c.2102-2103insA (p.N701Kfs*13)	NK	[54]
81	DD (3)	PJ, HSM	NK	Bedouin-Israeli	c.1211G >A (p.R404Q)	c.1211G > A (p.R404Q)	40	[47]
82	DD, DR (12)	PJ, HSM	NK	Bedouin-Israeli	c.1211G >A (p.R404Q)	c.1211G > A (p.R404Q)	35	[47]
83	DD, DR (24)	HSM	NK	Bedouin-Israeli	c.1211G >A (p.R404Q)	c.1211G > A (p.R404Q)	53	[47]
84	DD, DR (24)	HSM, PJ	NK	Bedouin-Israeli	c.1211G >A (p.R404Q)	c.1211G > A (p.R404Q)	46	[47]
85	DD, DR (18)	HSM, PJ	NK	Bedouin-Israeli	c.1211G >A (p.R404Q)	c.1211G > A (p.R404Q)	52	[47]
86	Hypotonia, abnormal gait (20)	HSM	NK	French	c.1211G >A (p.R404Q)	c.709C>T (p.237S)	64	[55,56]
87	DD (12)	HSM, PJ	NK	French	c.2324A>C (p.Q775P)	c.2324A>C (p.Q775P)	44	[55,56]
88	DD (10–12)	HSM, PJ	NK	French	c.1892T>G (p.M631R)	NK	42	[55,56]
89	Abnormal gait, speech problems (20–24)	HSM	NK	Tunisian	c.1553G>A (p.R518Q)	NK	60	[55,56]

HSM: hepatosplenomegaly, VM: visceromegaly, SM: splenomegaly, PJ: prolonged jaundice, DD: developmental delay, DR: developmental regression, M: male, F: female, NK: not known, Ref: reference.

**Table 3:** Range of neurological symptoms and signs. Patients selected from the total group on the basis of presence of more than one neurological symptom or sign. Patient number is linked to Table 1.

Patient No	Presenting Neurological Sign/Symptom (Age of Onset)	Developmental Progress	Ataxia, Abnormal Gait (Age of Onset)	Cataplexy (Age of Onset)	Seizures (Age of Onset)	VSGP (Age of Onset)	Dystonia (Age of Onset)	Dysphagia (Age of Onset)	Dysarthria (Age of Onset)	Spasticity (Age of Onset)
1	Swallowing difficulties, lack of motor coordination (3 m)	Psychomotor retardation				Yes		Yes		Yes
2	Severe encephalopathy with uncontrolled movements, ataxia, tremor, nystagmus (24 m)									
5	Hypotonia, DD (5 m)	Motor and cognitive deficits				Yes (9 m)	Yes	Yes		
6	Hypotonia, DD (6 m)	Motor and cognitive deficits				Yes (18 m)	Yes			
7	Hypotonia, DD (7 m)	Motor and cognitive deficits				Yes (24 m)	Yes		Yes	
8	Hypotonia, DD (9 m)	Motor and cognitive deficits								
9	Hypotonia, DD (9 m)	Motor and cognitive deficits							Yes	
10	Hypotonia, DD (10 m)	Motor and cognitive deficits				Yes			Yes	
11	Hypotonia, DD (12 m)	Motor and cognitive deficits						Yes	Yes	
12	Hypotonia, DD (12 m)	Motor and cognitive deficits					Yes		Yes	
13	Frequent falls, DD (12 m)	Delayed motor development								
14	Frequent falls, DD (18 m)	Independent walking at 18 m, language delay		Yes (30 m)						
15	Frequent falls, DD (12 m)	Independent walking at 12 m, slower intelligence progression, psychomotor regression		Yes (36 m)						
16	DD (20 m)	Delayed independent walk, slow motor development, psychomotor regression 24 m								
17	Frequent falls (15 m)	Motor regression 36 m		Yes (36 m)						
18	Frequent falls (20 m)			Yes						
19	DD (NK)	Developmental delay <24 m	Yes (<24 m)	Yes (24 m)	Yes (48 m)	Yes		Yes (60 m)	Yes	
20	DD (NK)	Developmental delay, never mobile, no swallowing problems		Yes (60 m)	Yes (70 m)	Yes (36 m)			Yes (<36 m)	
21	DD (NK)	Developmental delay, never mobile, no swallowing problems		Yes (60 m)	Yes (70 m)	Yes (36 m)			Yes (<36 m)	
22	DD (17 m)	Developmental delay 17 m, ataxia	Yes (48 m)	Yes (48 m)	Yes (60 m)	Yes (36 m)		Yes (60 m)	Yes	
23	DD (NK)	Developmental delay <18 m	Yes (<24 m)	Yes (35 m)				Yes (13 m)	Yes (<24 m)	
24	DD (NK)	No speech			Yes (41 m)	Yes (<18 m)		Yes (36 m)		
29	DR, ataxia (18 m)									
30	DD, ataxia (22 m)	Psychomotor regression								
31	DD (12 m)	Developmental delay at 1st year, cannot stand and walk at 19 m, tremor in upper limbs, no speech			Yes (30 m)					Yes (42 m)
32	Speech regression (18 m)	Early development was normal, walked independently at 14 m. Loss of speech at 18 m, cannot walk at 30 m, left hemiparesis, could not stand at 36 m, bedridden at 48 m				Yes			Yes	
33	Hypotonia (2m), DD (4 m)	2m hypotonia, poor sucking, developmental delay (poor head control at 4 m)								
35	Hypotonia, DD (12 m)	Psychomotor retardation								
37	Motor regression, spastic tetraparesis (12 m)	Motor regression								
51	Hypotonia (23 m)	Neurological regression 24 m								
55	DR (NK)	Developmental regression, intellectual disability								
56	DR (NK)	Developmental regression, intellectual disability								
57	DR (NK)	Developmental regression, intellectual disability, hearing impairment, visual impairment				Yes				
58	DR (NK)	Developmental regression			Yes					
59	DD, ataxia (24)	Mild global developmental delay				Yes (54 m)			Yes (54 m)	
75	DD (5)	Head control 5m, walking independently 24 m	Yes (30 m)			Yes (45 m)	Yes (45 m)	Yes (36 m)	Yes (36 m)	Yes (45 m)
76	DD, DR (9)	Developmental regression 9m, never walk, no speech	Yes	Yes	Yes	Yes		Yes		Yes
77	Hypotonia, DD (NK)	Mild psychomotor retardation, decreased muscular tone, walking difficulties								
78	Hypotonia, DD, Dystonia (5)	Able to sit: 18 m Never stand up or walk				Yes (8 m)				
82	DD, DR (12)									
83	DD, DR (24)									
84	DD, DR (24)									
85	DD, DR (18)									

DD: developmental delay, DR: developmental regression, VSGP: vertical supranuclear gaze palsy, M: male, F: female, m: months, NK: not known.

**Table 4:** *NPC1* mutations causing early infantile phenotype. Mutation–phenotype association.

		EISL Phenotype in Combination with the Following Mutations	Early Infantile Phenotype in Homozygous State	Early Infantile Phenotype in Combination with	Late Infantile Phenotype in Combination with the Following Mutations	Juvenile Phenotype in Combination with the Following Mutations
Deletions/Insertions	exon 1+promoter		X			
	c.385delT		X			
	c.960_961dup (p.A321Gfs*16)		X			
	p.C63fsX75		X			
	c.3578_3591 + 9del		X			
Missense Mutations	c.3614C>G (p.T1205R)	-c.1261C>T (p.Q421X)		-c.3614C>A (p.T1205K)	-c.1553G>A (p.R518Q)	
	c.3107C>T (p.T1036M)		X	-c.3557G>A (p.R1168H)	-c.3182C>T (p.I1061T)	-c.2861C>T (p.S954L)
	c.1553G>A (p.R518Q)			c.2795dupA	-Homozygous -c.1172A>G (p. E391G)	-c.3019C>G (p.P1007A) -c.494C>T (p.A165V)
	c.3557G>A (p.R1186H)			-c.3107C>T (p.T1036M) -c.826T>C (p.Y276H) -c.3614C>A (p.T1205K) -c.2196dupT (p.P733Sfs*9)	-c.1421C>T (p.P474L) -c.3019C>G (p.P1007A) -c.826T>C (p.Y276H) -c.3182T>C (p.I1061T) -c.2830G>A (p.D944N) -c.2196dupT (p.P733Sfs*9)	- c.2861C>T (p.S954L)
	c.1415T>C (p.L472P)		X		-Homozygous	
	c.3104C>T (p.A1035V)		X	-IVS23 +1G > A (c.3591+1G>A)		-NK -c.3019C>G (p.P1007A)
	c.3394G>C (p.A1132P)		X		-Homozygous	
	c.3503G>A (p.C1168Y)		X		-Homozygous	
	c.1211G>A (p.R404Q)	-Homozygous	X	-c.709C>T (p.237S)	-Homozygous	-c.1133T>C (p.Vl378A) -NK
	c.2740T>A (p.C914S)		X			
	c.3584G>T (p.G1195V)		X			
Splice Site Mutations	IVS23 +1G > A (c.3591+1G>A)		X	-c.3104C>T (p.A1035V) -c.3182T>C (p.I1061T)	-c.2090C>T (p.V697A)	
	c.3478-6T>A		X			
	IVS21-2delATGC		X			

**Table 5:** Demographic, clinical, and molecular features of early infantile disease with *NPC2* mutations.

Patient No	Presenting Neurological Sign/Symptom (Age of Onset in Months)	Visceral Symptoms	Ethnicity	Sex F/M	Allele 1	Allele 2	Age of Death (Month)	Ref.
1	DD (12)	HSM, PI	Czech	F	c.58G>T (p.E20X)	c.58G>T (p.E20X)	48 (RF)	[36]
2	NK	HSM, PI	Italian	NK	c.58G>T (p.E20X)	c.58G>T (p.E20X)	10 (RF)	[40]
3	NK	HSM, PI	Italian	NK	c.58G>T (p.E20X)	c.58G>T (p.E20X)	NK	[40]
4	DD, Hypotonia (12)	SM	Turkish	F	c.352G>T (p.E118X)	c.352G>T (p.E118X)	24 (RF)	[67]
5	Hypotonia	PJ, HSM, PI	Turkish	F	c.352G>T (p.E118X)	c.352G>T (p.E118X)	10 (RF)	[67]
6	Hypotonia, DD (2)	HSM, PJ, PI	Turkish	F	c.434T>A (p.V145E)	c.434T>A (p.V145E)	8 (RF)	[68]
7	Newborn Hypotonia (NK)	HSM, PJ, PI	Turkish	F	c.434T>A (p.V145E)	c.434T>A (p.V145E)	9 (RF)	[67]
8	Hypotonia (4)	SM	Turkish	NK	c.352G>T (p.E118X)	c.352G>T (p.E118X)	NK	[67]
9	Hypotonia (2)	SM, PJ, PI	Tunisian	M	c.436C>T (p.Q146X)	c.436C>T (p.Q146X)	4.5 (RF)	[65]
10	Hypotonia, DD (8)	PI, HSM	German	F	c.352G>T (p.E118X)	c.352G>T (p.E118X)	11 (RF)	[65]
11	DD (7)	PJ, PI	Algerian	M	c.58G>T (p.E20X)	c.58G>T (p.E20X)	19 (RF)	[64,69]
12	Hypotonia, abnormal gait (18)	HSM, PI	Turkish	F	c.199T>C (p.S67P)	c.199T>C (p.S67P)	Alive (45y)	[64]
13	DD (16)	HSM, PJ	Italian	NK	c.133C>T (p.Q45X)	c.133C>T (p.Q45X)	Alive (54)	[70]
14	Hypotonia, DD (1)	HM, PJ, PI	Sri-Lankan	NK	c.141C>A (p.C47X)	c.141C>A (p.C47X)	Alive (12)	[70]
15	Hypotonia (12)	HM, PI	Indian	M	c.82+2T>C (IVS1+2T>C)	c.82+2T>C (IVS1+2T>C)	NK	[71]
16	DD (12)	SM	Chinese	F	c.3G>C (p.M1I)	c.190+5G>A(IVS2+5G>A)	NK	[46]

HSM: hepatosplenomegaly, VM: visceromegaly, SM: splenomegaly, PJ: prolonged jaundice, DD: developmental delay, PI: pulmonary involvement, M: male, F: female, NK: not known, Ref.: reference.

**Table 6:** *NPC2* mutations causing early infantile phenotype. Mutation–phenotype association.

		EISL Phenotype in Combination with the Following Mutations	Early Infantile Phenotype in Homozygous State	Early Infantile Phenotype in Combination with the Following Mutations	Late Infantile Phenotype in Combination with the Following Mutations	Juvenile Phenotype in Combination with the Following Mutations
**Missense Mutations**	c.58G>T (p.E20X)	-Homozygous -c.27delG (p.L10Sfs*25)	X		-Homozygous	
	c.352G>T (p.E118X)	-Homozygous	X			
	c.434T>A (p.V145E)		X			
	c.133C>T (p.Q45X)		X			
	c.141C>A (p.C47X)		X			
	c.199T>C (p.S67P)		X			
	c.436C>T (p.Q146X)	-Homozygous	X			
	c.3G>C (p.M1I)			- c.190+5G>A(IVS2+5G>A)		
**Splice Site**	c.82+2T>C (IVS1+2T>C)	-Homozygous	X			
	c.190+5G>A (IVS2+5G>A)			-c.3G>C (p.M1I)		-Homozygous
